# Investigation of the halophilic PET hydrolase PET6 from *Vibrio gazogenes*

**DOI:** 10.1002/pro.4500

**Published:** 2022-12

**Authors:** Sebastian Weigert, Pablo Perez‐Garcia, Florian J. Gisdon, Andreas Gagsteiger, Kristine Schweinshaut, G. Matthias Ullmann, Jennifer Chow, Wolfgang R. Streit, Birte Höcker

**Affiliations:** ^1^ Department of Biochemistry University of Bayreuth Bayreuth Germany; ^2^ Department of Microbiology and Biotechnology University of Hamburg Hamburg Germany; ^3^ Computational Biochemistry University of Bayreuth Bayreuth Germany

**Keywords:** hydrolases, MD simulation, metagenome, PET degradation, PETase, polyethylene terephthalate, substrate interaction

## Abstract

The handling of plastic waste and the associated ubiquitous occurrence of microplastic poses one of the biggest challenges of our time. Recent investigations of plastic degrading enzymes have opened new prospects for biological microplastic decomposition as well as recycling applications. For polyethylene terephthalate, in particular, several natural and engineered enzymes are known to have such promising properties. From a previous study that identified new PETase candidates by homology search, we chose the candidate PET6 from the globally distributed, halophilic organism *Vibrio gazogenes* for further investigation. By mapping the occurrence of *Vibrios* containing PET6 homologs we demonstrated their ubiquitous prevalence in the pangenome of several *Vibrio* strains. The biochemical characterization of PET6 showed that PET6 has a comparatively lower activity than other enzymes but also revealed a superior turnover at very high salt concentrations. The crystal structure of PET6 provides structural insights into this adaptation to saline environments. By grafting only a few beneficial mutations from other PET degrading enzymes onto PET6, we increased the activity up to three‐fold, demonstrating the evolutionary potential of the enzyme. MD simulations of the variant helped rationalize the mutational effects of those mutants and elucidate the interaction of the enzyme with a PET substrate. With tremendous amounts of plastic waste in the Ocean and the prevalence of *Vibrio gazogenes* in marine biofilms and estuarine marshes, our findings suggest *that Vibrio* and the PET6 enzyme are worthy subjects to study the PET degradation in marine environments.

## Abstract 2

PDB Code(s): 7Z6B;

## 1 INTRODUCTION

Plastic pollution, which is considered an urgent current threat, is a consequence of the massive popularity of plastic materials starting in the 1950s paired with enduring inadequate waste management worldwide. While the world plastic production has grown to enormous 415 million tons in 2016,^1^ rates for recycling and incineration are still low, with over 50% of the plastic waste being discarded despite large improvements over the last decades.^2^ Within this setting, microbiological research has focused on identifying plastic‐active enzymes in the last decade. For some polymers enzymes have been reported acting on the polymers. These include ester‐based polyurethanes (PU), and plastic polyethylene terephthalate (PET) polyesters, which in principle can be cleaved by members of the extensive class of hydrolases.^3^ In the scope of plastic pollution, hydrolases with activity on the commodity PET came to the fore. Today, there are 40 PET‐active enzymes known.^3^ They are affiliated with four bacterial and two fungal phyla. The first PET degrading enzyme TfH was reported in 1998 by Kleeberg et al., which was isolated from *Thermobifida fusca* belonging to the order of *Actinomycetales*.^4^, ^5^ As members of this order are common in the context of plant material degradation, including cutin,^6^ many PET degrading enzymes identified were found from bacteria of this phylum, including prominent TfCut2, Thc_Cut1, Est119, and LCC.^7^, ^8^, ^9^, ^10^, ^11^ Over the last years, protein engineering has been applied to increase activity and thermostability of these enzymes, enabling future recycling applications. Recently a number of exciting articles have been published reporting improved catalytic activities of either IS, PET2, or LCC.^12^, ^13^, ^14^, ^15^, ^16^, ^17^, ^18^, ^19^ A variety of strategies targeting sequence‐based knowledge, substrate‐binding or the catalytic mechanism have been used including machine‐learning or directed evolution that resulted in enhanced degradation capabilities thereby emphasizing the high potential of engineered PET‐hydrolases. In fact, Tournier et al. already presented a proof of concept for enzymatic PET recycling employing engineered variants of LCC.^20^

In 2016, the discovery of *Ideonella sakaiensis* as a bacterium that can exploit PET as sole energy and carbon source attracted particular attention.^21^ This ability is facilitated by a two‐enzyme system that consists of a cutinase‐like enzyme called PETase (IsPETase) undertaking the coarse degradation whose main product mono‐(2‐hydroxyethyl)‐terephthalate (MHET) is further hydrolyzed by the second enzyme MHETase. The latter is related to feruloyl esterase and shows a comparably high specificity toward its substrate MHET,^22^ and increases PET hydrolysis rates of IsPETase when used together.^23^

In contrast to most previously characterized enzymes, IsPETase shows decent activity at temperatures of 30–40°C, implying the possibility for substantial PET degradation in the environment. To evaluate this, the prevalence of such enzymes must be analyzed, and their actual degradation activity on PET subsequently investigated. Accordingly Danso et al. conducted a bioinformatic search employing a hidden Markov model (HMM) approach trained with the structure–function relationships from known PET hydrolase to identify new potential enzymes in genomic samples from various environments.^24^ They identified several potential enzymes, and PET hydrolase activity was measured among others for PET6.^24^ This enzyme is found in the proteobacteria *Vibrio gazogenes* from the genus *Vibrio*, whose members are ubiquitously present in saline and marine environments.^25^ A search in the PAZy database identified two additional marine‐derived enzymes originating from *Pseudomonas* aestusnigri and from *Marinobacter* sp.^26^, ^27^ Of particular interest in the scope of plastic pollution is their prevalence in estuaries, salt marshes, and in the plastisphere, which describes the microbial environment around plastic particles.^28^, ^29^, ^30^, ^31^ As there are million tons of plastic particles in the oceans and rivers as a main entrance path for plastic in the environment^32^ mouthing in estuaries and neighboring salt marshes, PET6 is an interesting candidate to investigate its PET degradation potential. In particular, ecosystems like estuaries and salt marshes combine the reported prevalence of *Vibrio* species and high concentrations of plastics and microplastics.^33^, ^34^

Of the variants found and initially characterized in the study by Danso et al.,^24^ PET6 of *Vibrio gazogenes* is a representative that, in addition to its described PETase activity, promises large‐scale occurrence. It is well known that *Vibrio* species are found nearly everywhere, especially in marine environments, where they play a critical role in carbon and nitrogen cycling.^35^ Furthermore, atmospheric warming is enhancing the global occurrence of this genus.^36^ In several studies describing the microbial community on plastic debris (“plastisphere”), the genus *Vibrio* was found to be the most abundant taxon able to colonize PET (40%), polyethylene (PE, 30%) or polypropylene (PP, 33%).^37^, ^38^, ^39^ Moreover, several pathogenic Vibrios have been identified on the aforementioned plastics recovered from oceans, but also on polystyrene (PS) and polyvinyl chloride (PVC; see table 1 in Bowley et al.^40^). To which extent these *Vibrios* could be involved in degradation of these plastics remains yet unknown. In this study, we characterize PET6 from *Vibrio gazogenes* in detail and evaluate its potential for PET degradation in near‐realistic saline conditions. This enables insights into the adaptation of this enzyme toward its environment and allows for a rough estimation of whether members of the genus *Vibrio* might facilitate PET degradation in the environment.

## 2 RESULTS AND DISCUSSION

### 2.1 Global prevalence of PET6 homologs in the *Vibrio* genus

We aimed at identifying other putative PET‐degrading Vibrios by performing a BLASTp search against NCBI's non‐redundant database filtered for *Vibrio* spp. (taxid 662). This resulted in eight hits with full coverage and more than 79% sequence identity compared to the PET‐hydrolyzing PET6 in *V. gazogenes*, *V. spartinae*, *V. ruber*, *V. zhugei*, and *V. palustris* (Table S1). All of these species were isolated from marine or saline environments,^41^, ^42^, ^43^, ^44^ implying possible adaption to saline conditions.

**FIGURE 1:**
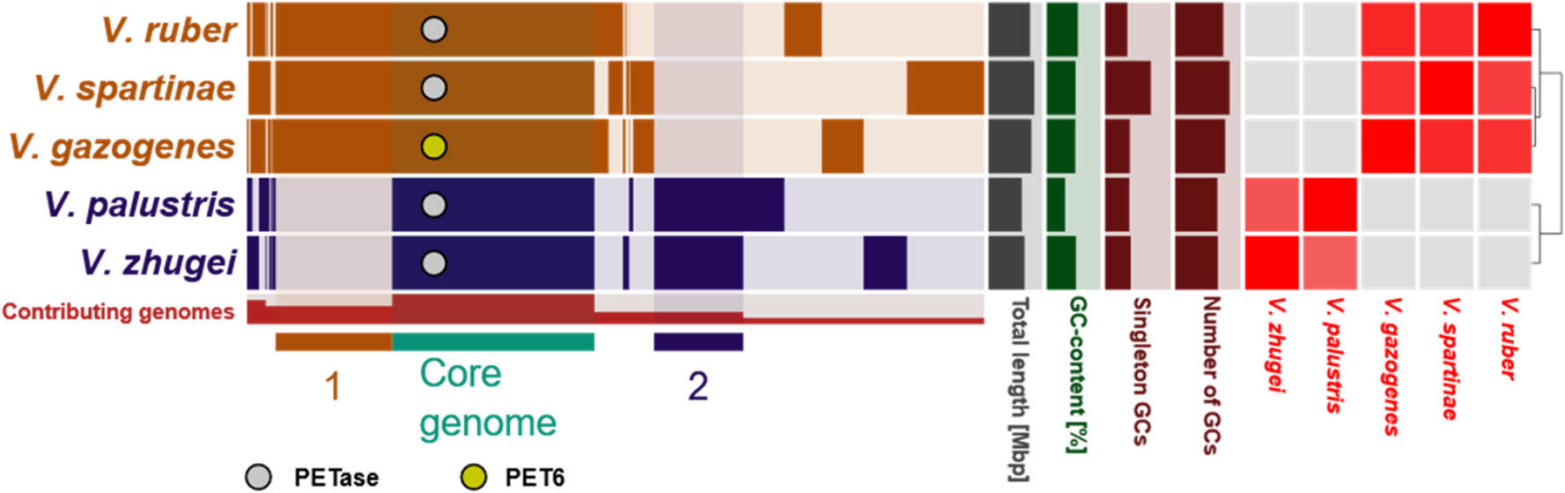
Pangenomic analysis of five PETase‐containing *Vibrio* genomes. The analysis includes 6,651 GCs involving 19,117 individual gene calls. The five first tracks represent protein‐coding GCs of individual genomes organized by ANI (red squares and cladogram) of the aligned fraction. Presence of a GC in a genome is indicated in dark colors, absence in light. The core genome of the analyzed *Vibrio* genomes is indicated in turquoise. Further GCs specific of each of the two clades are indicated in the color of the clade (1 and 2). The red track shows the number of genomes harboring a defined GC. Other genome statistics (from left to right: total length [0–6 Mbp], GC content [30–50%], number of singleton GCs [0–1,000] and number of GCs [0–5,000]) are indicated as additional bar charts on the right. The GCs containing PETase‐coding genes are marked with a circle for each species. PET6 is highlighted in yellow. ANI, average nucleotide identity; GCs, gene clusters; PET, polyethylene terephthalate

A pangenomic analysis of one representative genome of each PETase‐containing *Vibrio* spp. revealed a core genome of 1,824 gene clusters (GCs; Figure 1), which included all putative PETases. The accessory genome ranged from 1,392 to 2,321 GCs for the analyzed isolates. Due to their high sequence similarity, all five PETases were allocated in the same GC. According to Average Nucleotide Identity (ANI) analysis, the analyzed Vibrio species belong to two distinct lineages, where additional 1,050 and 805 GC are shared exclusively between the members. This strongly suggests the horizontal transfer of PETase genes within the genus (Figure 1). The “plastisphere” of both aquatic and soil environments has been identified as a “hotspot” for gene transfer.^45^, ^46^ This is further supported by the extended analysis in Figure S1, including 28 additional genomes from representative pathogenic and non‐pathogenic Vibrios that do not contain genes coding for PETases. ANI analysis revealed the presence of two main *Vibrio* clades (I and II), where all Vibrios with predicted PET‐degrading ability were allocated in Clade II. Again, the PETase‐coding *V. gazogenes*, *V. spartinae*, and *V. ruber* clustered closely together, but *V. palustris* and *V. zhugei* appear closer related to *V. tritonius* and *V. nitrifigilis*.

Taken together, these results indicate that some Vibrios are theoretically equipped to act on PET, making *Vibrio*‐mediated PET hydrolysis in marine ecosystems conceivable. The next step in the classification of PET6 and related enzymes in the *Vibrio* genus is a detailed analysis including its activity. Here, we use PET6 as a prototype.

### 2.2 Characterization of PET6 activity

**FIGURE 2:**
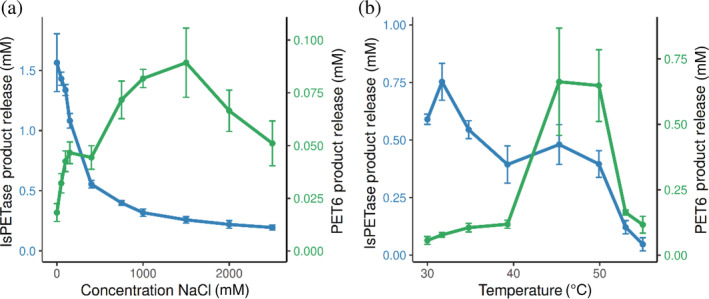
Activity of PET6 and IsPETase on PET at varying temperatures and salt concentrations. PET6 (green line) and IsPETase (blue line) were incubated for 18 hr at a concentration of 2 μM and 20 nM, respectively. (a) The effect of salt concentrations on the two enzymes varied between 0 and 2,500 mM (measured in 96‐well reaction plates at 30°C). (b) Different incubation temperatures in the range of 30 and 55°C were tested (measured in coated PCR tubes) with the sodium chloride concentration fixed at 50 mM. PET, plastic polyethylene terephthalate

PET6 was expressed heterologously in *Escherichia coli* and purified to homogeneity using an encoded histidine tag. After purification, we first validated and characterized the enzyme's capabilities to degrade PET. Here, we employed our previously developed assay platform for PET degradation, which works with a PET‐coating inside standard lab consumables as substrate.^47^ The coating was prepared from post‐consumer PET bottles to achieve a near‐realistic substrate with a crystallinity of around 10%. Besides the general capacity for PET degradation, the enzyme's individual optima regarding temperature and ionic strength were investigated. Since PET6 was derived from a marine organism, the experimental setup for examining the enzyme's activity in the presence of salt covered sodium chloride concentrations between 25 and 2,500 mM (Figure 2a). Similarly, we determined the optimum temperature in the range from 30 to 55°C (Figure 2b). As a reference, the well‐studied IsPETase was included for both quantitative benchmark and qualitative comparison of the determined optima. The activity was determined by UHPLC summing up TPA, BHET, and MHET to the total product release. As IsPETase showed substantially higher activity at the given conditions, the concentration was adjusted to fit the substrate limits of the assay. Therefore, IsPETase was used at 20 nM while PET6 was employed at 2 μM.

The results indicate that IsPETase has a relatively broad temperature range for activity with an optimum around 30°C, whereas PET6 shows a distinct optimum between 45 and 50°C. The different behavior of the two enzymes is even more noticeable when looking at the impact of ionic strength on the PET degradation activity. While PET6 shows little activity at low salt concentrations, the product release increases rapidly with an optimum between 1 and 1.5 M NaCl. In contrast, IsPETase displays the opposite behavior toward salt with an optimum at low salt and rapidly decreasing activity with rising ionic strength.

### 2.3 Structural analysis

**FIGURE 3:**
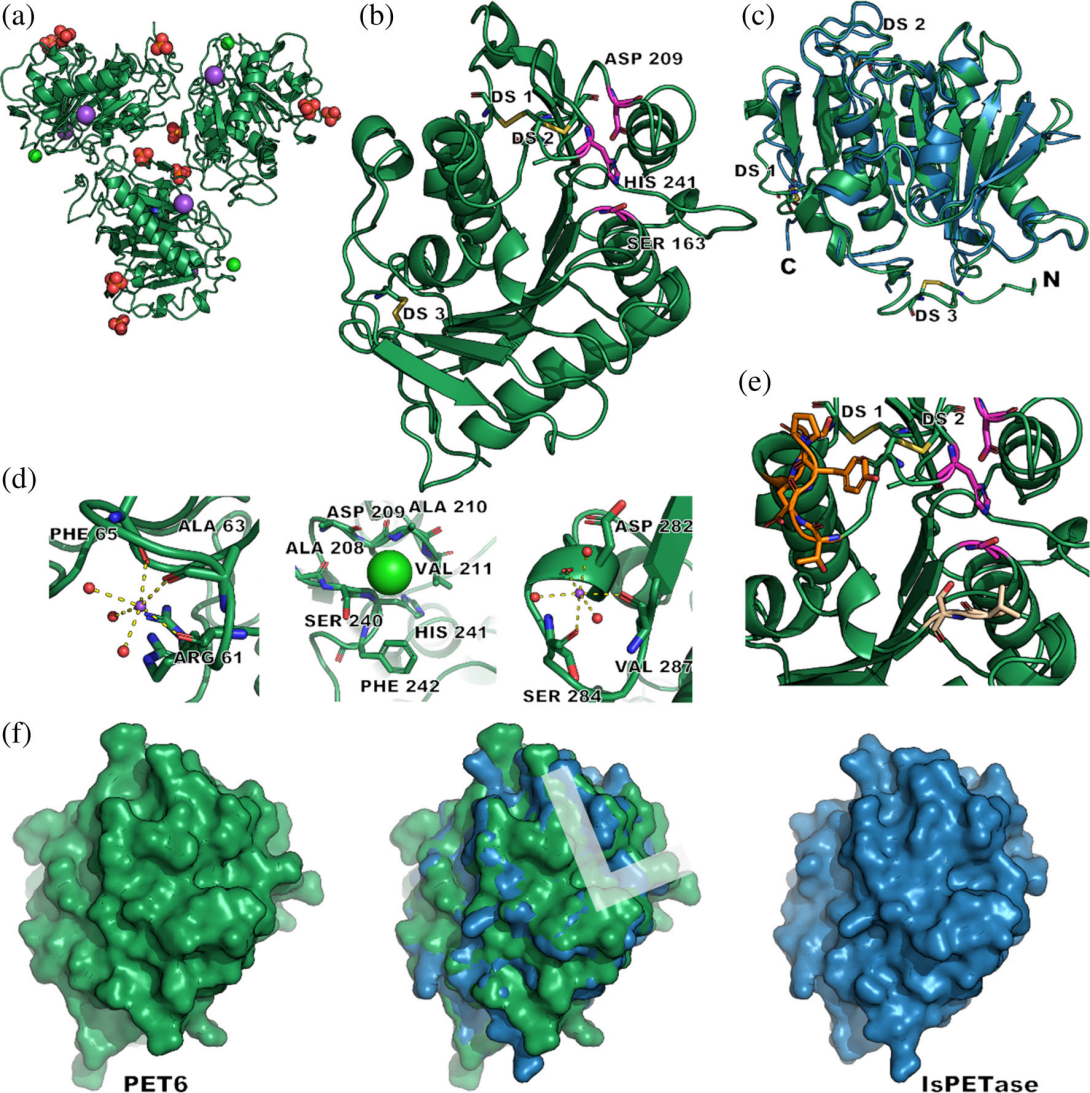
Crystal structure of PET6 (green) and comparison to IsPETase (blue). (a) Shows the asymmetric unit of the PET6 crystal structure (green, PDB ID 7Z6B) containing three protein molecules; ions are depicted as colored spheres including phosphate (orange‐red), sodium (purple), and chloride (neon green). (b) Displays a single chain, with the catalytic triad (Ser‐His‐Asp) highlighted in pink; the three disulfide bonds are labeled DS1‐3. (c) Illustrates how the superimposed structures of PET6 and IsPETase (5XJH) have only a few deviations in loops and termini. (d) Highlights ion binding with the two sodium ions (violet) being coordinated by three carbonyl oxygens and three water molecules, while chloride (neon green) is located in a small pocket. The positions of subsequent mutations are shown in (e) with the loop region (Ex‐Loop) in dark orange and two residues for the double mutant VSTA in beige in proximity to the active site colored in pink. (f) Compares the surface of the two proteins with a focus on the putative substrate‐binding site approximated as L shape in the superimposed structure.

Next, we aimed to investigate if the differences between IsPETase and PET6 activity are reflected on a molecular level. Thus, crystal trials were set up and an X‐ray structure of PET6 was obtained. The solved structure (PDB ID 7Z6B) shows three protein entities in the asymmetric unit. The maps allowed the modelling of three phosphates, two sodium and one chloride ion that are symmetrically arranged around each chain (Figures 3a and S2).

As indicated by the sequence similarity to other PET degrading enzymes, PET6 can be identified as an enzyme of the alpha/beta hydrolase fold. The characteristic topology of this class features eight beta‐strands connected with alpha‐helices and a conserved arrangement of the catalytic triad (Ser163, Asp209, and His241), with all of them present in PET6 (Figure 3b,c). As observed in other PETase crystal structures, all these enzymes are highly alike, with only minor differences in loop regions. The pseudo symmetry indicated by the crystallographic parameters is also reflected in the asymmetric unit of the solved structure. The arrangement of the three protein chains, including surrounding and bound ions, is highly symmetric (Figure 3c). Consequently, the chains show an all‐atom RMSD of only 0.61–0.68 Å among each other. PET6 features three disulfide bonds labeled DS 1–3 (Figure 3b,c,e). DS 1 is located close to the C‐terminus, formed between residue 277 and 294, and conserved in all cutinases. Therefore, it was included in the HMM search model from Danso et al.^24^ The second disulfide bond DS 2 connects residue 206 and 243 and is located close to the active site. This disulfide bond position has been mainly described for fungal cutinases but is also found in IsPETase. DS 3 is located near the N‐terminus between residue 27 and 30 and is not commonly described for PET hydrolases or cutinases.

The presence of three coordinated ions per chain in the asymmetric unit is of particular interest. Binding sites for divalent cations such as Ca^2+^, Zn^2+^, and Mg^2+^ have been described for several cutinases and PET hydrolases, showing stabilizing effects and increasing activity when bound.^48^ PET6, on the contrary, shows binding of monovalent ions with one sodium ion coordinated by the carbonyl oxygens of the residues Arg 61, Ala 63, and Phe 65; the typical octahedral coordination is completed by three water molecules (Figure 3d). The same set up is repeated for a second sodium ion, interacting with the residues Asp 282, Ser 284, and Val 287. A chloride ion can be modeled in a shallow pocket created by the residues Ala 208, Asp 209, Ala 210, Val 211, Ser 240, His241, Phe 242. Comparison of these ion binding sites with other cutinase structures revealed the same chloride binding site in Thc_Cut1 (PDB‐ID: 5LUI, 2× Mg^2+^, 3× Cl^−^) and Cut190 (PDB‐ID: 4WFK). The latter further shares the ion binding site around the residues 61, 63, and 65 of PET6 (Figure 3d) but coordinates a Ca^2+^ instead. This behavior toward monovalent ions might be a result of adapting to saline environments with high sodium chloride concentrations. Furthermore, it hints at how salt concentrations might impact the enzyme's activity.

Due to the similarity of IsPETase and PET6 (Figure 3c), the resulting shape and surface are also highly alike (Figure 3f), with slight differences in the loop regions and positioning of side chains. The predicted binding site of the PET strand for IsPETase is a very distinct shallow binding groove on both sides of the active site. Docking studies conducted by Joo et al. proposed an L‐shaped binding pose.^49^ A part of this binding site described by the authors is formed by a series of six residues, which are referred to as the extended loop. The surface of PET6 shows a similarly shaped binding groove but with one knob blocking the upper leg of the hypothetical L‐binding site, as visible in the figure of the superimposed surfaces (Figure 3f). This knob originates from Tyr248, while IsPETase's corresponding residue within the extended loop region is the smaller asparagine^49^ (Figure 3e). The B‐factors of Tyr248, including the sidechain, show no abnormalities compared to the neighboring residues. Thus, there is no sign of particular flexibility that might indicate a flipping out movement. Consequently, this tyrosine might comprise an obstacle for an analogous binding mode to the one proposed for IsPETase and, therefore, might reduce activity. Yet the mode of this L‐shaped binding has also been questioned, mainly asking whether the PET substrate would take on the necessary conformation for that binding mode.^50^

### 2.4 PET6 variants

**FIGURE 4:**
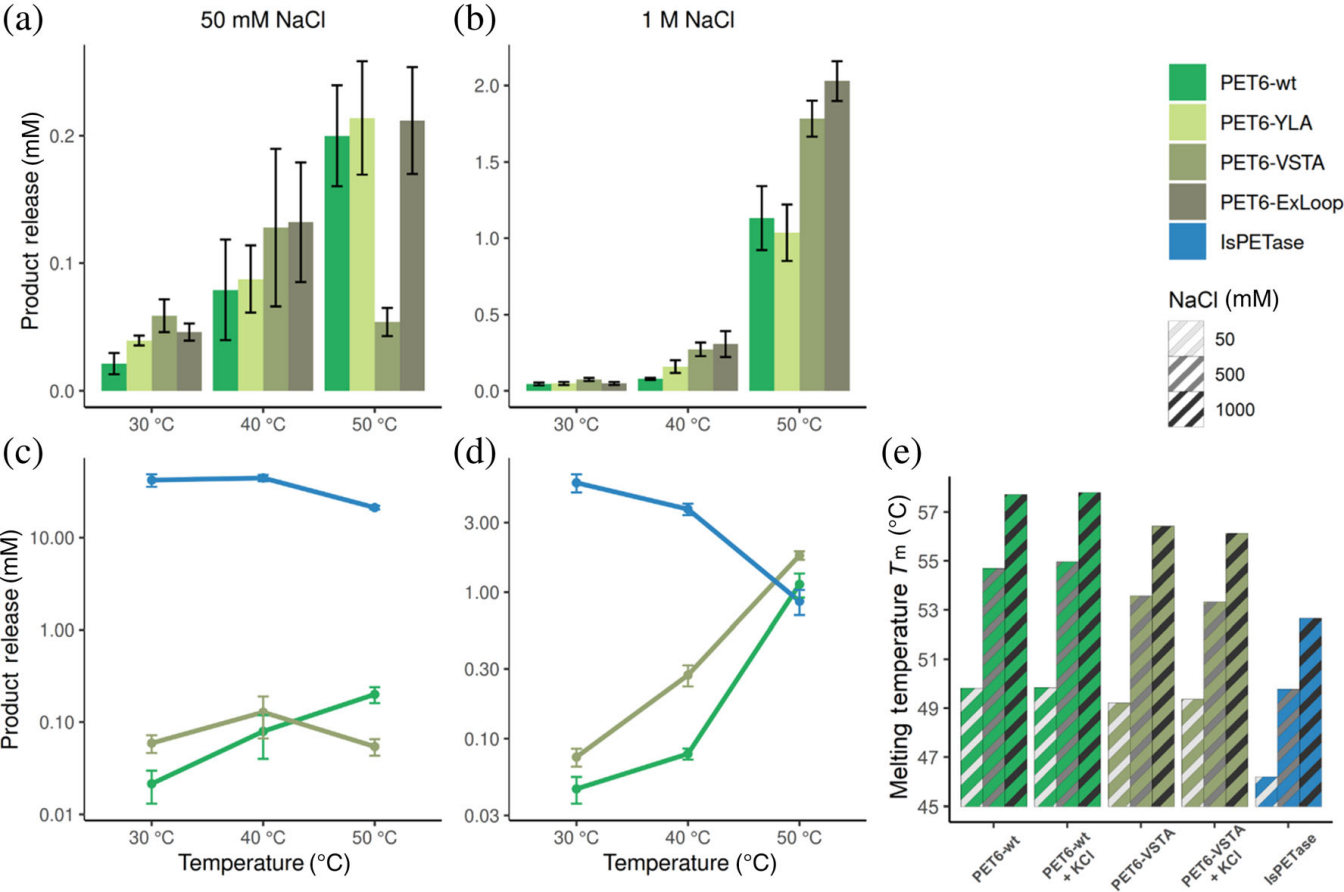
Performance of PET6 variants compared to IsPETase at varying temperatures and salt concentrations. PET6 variants (green) were used at a concentration of 2 μM and IsPETase (blue) at 200 nM and incubated for 18 hr. Therefore, the total product release of IsPETase was extrapolated by a factor of 10 to obtain comparable results within the activity experiments (a–d). The performance of the PET6 variants at different temperatures is compared in (a) at 50 mM and (b) at 1 M salt. The same data is shown in (c) and (d) for PET6‐wt and PET6‐VSTA with IsPETase for comparison; therefore, the *y*‐axis is log10 scaled to account for strongly varying performance levels. (e) Shows the analysis of *T*_m_ for PET6‐wt, PET6‐VSTA, and IsPETase at varying salt concentrations with DSC. The *T*_m_ of the enzymes was determined at 50, 500, and 1,000 mM NaCl as indicated in the hatching; the two PET6 variants were further characterized under the same conditions with additional 10 mM KCl. DSC, differential scanning calorimetry

To explore the evolutionary potential of PET6, we tested some mutants of the enzyme. The first variant targets the tyrosine in the extended loop of PET6 that was revealed in the crystal structure. To check if this tyrosine hinders substrate‐binding based on the mode described by Joo et al.,^49^ we mutated this residue to the less bulky alanine (PET6‐YLA, mutation Y248A). In a second variant, the entire extended loop (residues 246–251, sequence TGYPSE) was exchanged with the SGNSNQ‐sequence from IsPETase (PET‐ExLoop). Joo et al.^49^ had also reported the conservation of Thr88 and Ala89 (IsPETase numbering), which we transferred to PET6, changing the corresponding valine to threonine and serine to alanine, creating PET6‐VSTA (mutations V91T, S92A). The three variants were benchmarked against each other and IsPETase as reference. In this comparison, two salt concentrations (50 and 1,000 mM NaCl), and three different temperatures (30, 40, and 50°C) were tested. Due to the significantly higher activity of IsPETase compared to PET6, IsPETase was employed in a lower concentration to fit within the experimental substrate limits; eventually, the results were extrapolated accordingly by a factor of 10 to compare them to the PET6 variants (Figure 4c,d).

At the lower salt concentration of 50 mM, all PET6 variants show considerably lower activity (Figure 4a). Yet, there is a trend that introduced mutations lead to improvements in activity, especially at 30°C. At higher temperatures, the increase in activity is less significant or entirely within the error margin as for 50°C. The variant VSTA stands out, showing the best improvements but for 50°C, where the performance breaks down dramatically to a total product release of 0.05 mM compared to 0.2 mM of the other variants, including PET6‐wt. At 1 M salt, the PET degradation is, as expected, much higher for all PET6 variants (Figure 4b), but especially at 40 and 50°C, the introduced mutations led to a significant increase in performance. In particular, this is true for PET6‐ExLoop and VSTA at 40°C where the total product release reaches 0.31 ± 0.06 and 0.27 ± 0.05 μM, respectively, compared to 0.08 ± 0.01 μM for the wild type. Somewhat less improvement can be seen at 50°C, where PET6‐ExLoop and PET6‐VSTA outperform PET6‐wt by 79 and 58%, respectively. In contrast, the single mutant PET6‐YLA, reconstructing the aforementioned L‐shaped binding site by removing the tyrosine as an obstacle in the binding groove, shows only a slight improvement in all conditions. However, the exchange of the whole extended loop improved the PET degradation performance, especially at 40–50°C and 1 M salt, suggesting a more complex contribution of these residues. Given these results, PET6‐VSTA is an appealing candidate for further experiments, for one because of its massive boosts in activity with only two mutations compared to the wild type, and for the other due to its surprising performance drop at 50°C at low salt.

When we compare these results of PET6‐wt and PET6‐VSTA with IsPETase, in the presence of 50 mM NaCl, IsPETase is magnitudes more active at all temperatures with an extrapolated total product release between 21 and 45 μM in contrast to 0.02–0.2 μM for PET6‐variants (Figure 4c). As seen in previous experiments (Figure 2b), the performance of IsPETase decreases at temperatures around 50°C (Figure 4c). At 1 M sodium chloride, IsPETase's performance decreases to a total product release of 5.6 μM at 30°C, which is further reduced with rising temperatures. This opposite temperature preference of PET6 and IsPETase culminates at 50°C, where PET6‐VSTA and PET6‐wt take the lead with 1.8 and 1.1 μM product release over IsPETase with 0.9 μM after extrapolation. This proves a decent PET degradation potential of PET6 under appropriate conditions and categorizes it as PET hydrolase according to Kawai et al.^51^

In general, these results suggest that temperature and thereby protein stability in combination with salt might be crucial for PET6‐wt activity, as well as for its variants and IsPETase. Therefore, DSC measurements were carried out for PET6‐wt and PET‐VSTA at 50, 500, and 1,000 mM NaCl to determine the melting temperature and investigate this interplay.

The analysis shows that higher salt levels generally stabilize the proteins, for example, PET6‐wt gains around 7°C in *T*_m_ from 49.8 to 57.7°C (Figure 4e). The same applies in principle for the PET6‐VSTA variant, but the two introduced mutations show a destabilizing effect accounting for a 0.5–1°C decrease in *T*_m_ compared to PET6‐wt at corresponding salt levels. Nevertheless, there is an evident trend in how PET6 benefits from improved stability at higher salt concentrations. Furthermore, the data offers a possible explanation as to why the performance of PET6‐VSTA breaks down at 50°C and low salt in contrast to the wildtype. Both enzymes are employed near or even above their *T*_m_ in this condition. However, the small additional destabilization by about 0.6°C upon the mutations in PET6‐VSTA might be sufficient to cause this dramatic activity loss.

Interestingly, the stability of IsPETase is also increased with higher salt concentrations from 46.2°C at 50 mM to 52.7°C at 1 M salt. However, this enhanced stability does not translate to higher activity (Figure 4). Once more, this demonstrates the close relation between PET6 performance and ionic strength, which raises the question, whether the type of monovalent ions plays a role. A natural candidate, besides sodium and chloride, is potassium. It is not only a typical component of seawater with high similarity to sodium but also present in the successful crystallization condition containing sodium‐potassium phosphate. Compounds in crystallization conditions often have stabilizing effects on the protein, eventually promoting crystallization. Another series of DSC measurements were conducted to test whether 10 mM potassium chloride has a stabilizing effect. However, there is no clear difference in the measurements between corresponding conditions (Figure 4e). The effect of potassium ions on the activity itself was tested in an experimental setup with 1 M salt as a base to minimize the relative increase in total ionic strength upon adding 10 mM potassium chloride. After incubation at 50°C for 18 hr the total product release of PET6‐wt increased about 10% from 476 ± 22 μM to 524 ± 33 μM, while rising by 8% for PET6‐VSTA from 745 ± 47 μM to 810 ± 86 μM. Despite the high standard deviation, these results strongly suggest a positive impact of potassium ions. Under the given conditions, 10 mM KCl corresponds only to a rise of about 1% of the total ionic strength, making it unlikely that this performance increase can only be attributed to the increased ion concentration in general. Additionally, divalent ions including Ca^2+^, Co^2+^, Cu^2+^, Mg^2+^, Mn^2+^, Ni^2+^, and Zn^2+^ have been tested in a concentration of 10 mM, but no increase in activity could be detected. This emphasizes the preference of PET6 toward monovalent ions and their specific impact on its activity in contrast to other cutinases and their interaction with divalent metal ions.

### 2.5 Comparison of the binding modes of a PET tetramer to PET6‐wt and to PET6‐VSTA

**FIGURE 5:**
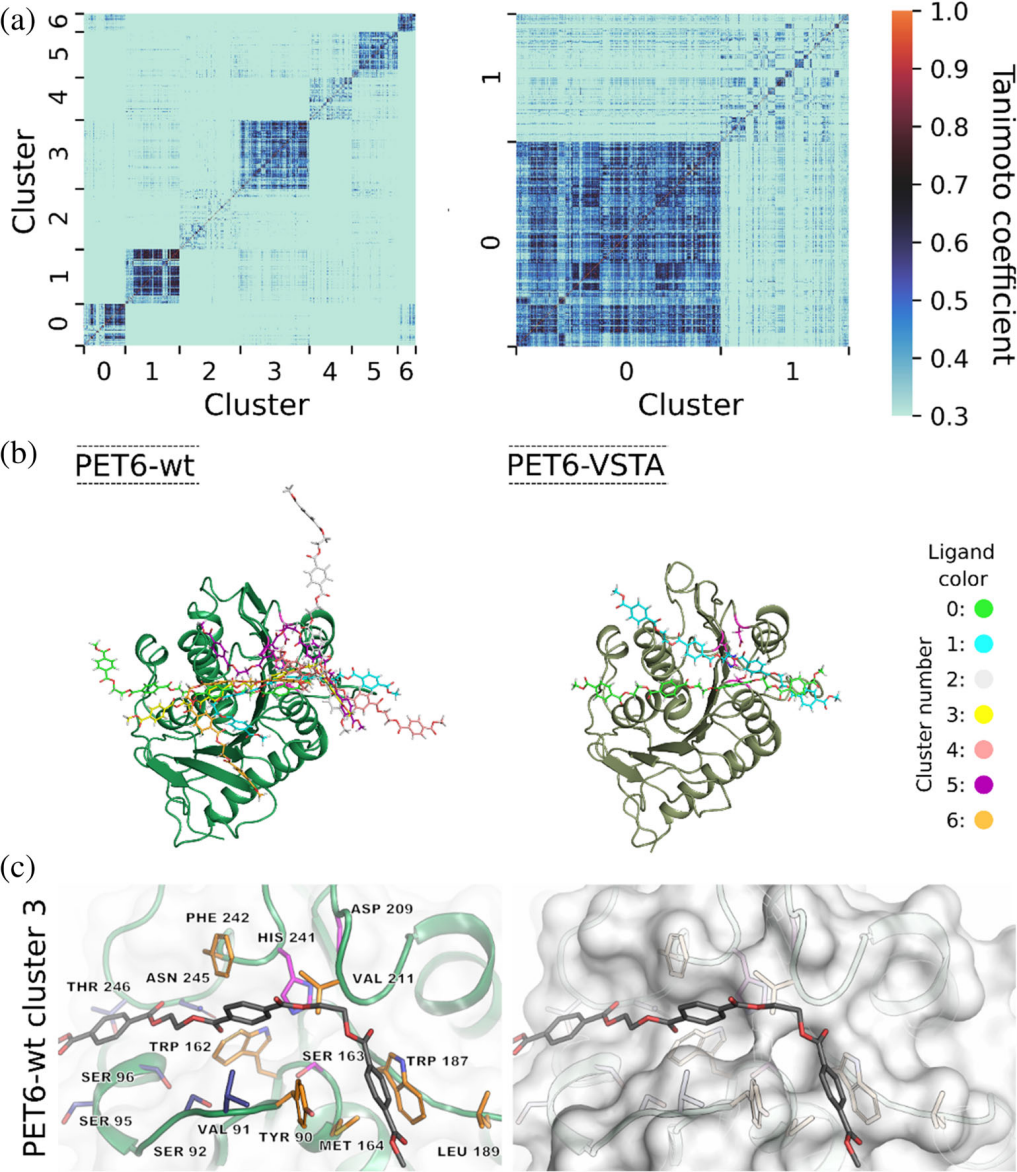
Binding modes of a PET tetramer in complex with PET6‐wt and PET6‐VSTA. (a) Tanimoto similarity matrices clustered according to similar interaction patterns representing clusters of different binding modes. (b) Superimposition of representative structures of the identified binding modes corresponding to the clusters in (a). (c) Detailed representation of residues frequently involved in enzyme–ligand interaction for PET6‐wt. The left side shows the enzyme in cartoon representation with the interacting residues as well as the catalytic triad (Ser163, Asp209, and His241) depicted in sticks. The residues colored in orange are involved in interactions in both PET6‐wt and PET6‐VSTA, while those shown in blue are additionally relevant in the context of PET6‐VSTA; the catalytic triad is marked in purple. The same perspective is shown on the right but with a less transparent surface to visualize the positioning of the ligand within the binding region

Because of the observed differences between PET6‐wt and PET6‐VSTA we set out to model the interactions with the polymer. For this we employed a PET tetramer as model substrate in molecular dynamics (MD) simulations. The parameters were set to 323 K and a total ionic strength of 1,050 mM representing the high salt condition where PET6‐wt showed the best performance in experiments. For each complex, several trajectories comprising a total of 400 ns were analyzed to calculate protein–ligand interaction fingerprints and binding modes using the analysis tool ProLIF.^52^ We analyzed the binding of the PET tetramer to PET6‐wt and PET6‐VSTA using cluster analysis on the basis of the Tanimoto coefficient using *k*_Means_ with the Silhouette score (Figure 5). A single dot represents a similarity of the binding motifs (the darker the dot, the more similar are the binding motifs). In the Tanimoto similarity matrices (Figure 5a), we observe clusters with different size and different specificities. All clusters define energetic minima on the energy landscape. Larger clusters represent deeper energy minima. The higher the average Tanimoto coefficient in the cluster, the more similar is the binding motif in the cluster, which can be interpreted as a higher binding specificity of the substrate to the protein.

For PET6‐wt, we identified seven clusters with different binding modes (Figure 5a,b). The clusters 0, 1, 3, 5, and 6 represent structurally well‐defined energetic minima. The clusters 2 and 4 are less specific. Thus, in PET6‐wt, we found several minima of which five are relatively specific but nevertheless they differ structurally among each other. In contrast to PET6‐wt, we identified only two clusters for PET6‐VSTA. Cluster 0 is large and represents a structurally specific and strongly pronounced energetic minimum, whereas cluster 1 is less specific. Hence, the binding of the PET tetramer to PET6‐VSTA is more specific than the binding to PET6‐wt. Nevertheless, PET6‐wt and PET6‐VSTA show both that the substrate interacts favorably in the region around the active site.

Binding of PET involves the same residues in PET6‐wt and PET‐VSTA, namely Tyr90, Trp162, Met164, Trp187, Leu189, Val211, and Phe242 (Figure 5c). These residues define a region, which is approximately binding two units of the PET tetramer. A further frequent contact with the PET tetramer is located around Ser95 and indicates a potential binding region for another PET unit. In that region, we further see pronounced interactions with Ser96, Asn245, and Thr246 for PET6‐VSTA, which are rather sparse for PET6‐wt. This finding is in line with the defined interactions of a longer portion of the PET tetramer with PET‐VSTA. The MD trajectories of the two enzymes indicate a dominance of π‐stacking contacts between the PET tetramer and the respective enzyme. The share of frames within the trajectories, which show π‐stacking interactions is higher for the complex of PET6‐VSTA with the PET tetramer (93 ± 2%) compared to the complex of PET6‐wt with the PET tetramer (78 ± 11%) reflecting a more defined interaction in the complex of PET6‐VSTA. In addition to the more defined binding of the substrate, we observed a higher contact frequency between the ligand and the catalytic histidine (His241) for PET6‐VSTA (64 ± 14%) compared to PET6‐wt (18 ± 27%), which is beneficial for optimal binding to the active site. These observed differences are a consequence of the introduced mutations Val91 to threonine and Ser92 to alanine.

In PET6‐VSTA, the hydroxyl group of Thr91 is frequently close to a π‐system of the terephthalate units of the PET tetramer indicating potential OH–π interactions. The distance value at the maximum of the observed distribution is 3.5 Å (Figure S3, maximum of the estimated probability density function), which is exactly the ideal value found in the literature.^53^, ^54^ In addition, we observe a hydrophobic contact between Cβ of Ala92 and an ethyl linker of the PET tetramer. Interestingly, the distance between the hydroxyl group of Thr91 and the Cβ of Ala92 (5.1 Å, Figure S3) is about the distance of the π‐system of a terephthalate unit to the ethyl linker in PET. This specific interaction is only possible in PET6‐VSTA and not in PET6‐wt, which explains a more specific interaction with the PET substrate in the mutant.

In conclusion, the simulations show that the exchange of Val91 and Ser92, which are present in all natural PET6 variants, to threonine and alanine, respectively, enhances the interactions of the PET tetramer with PET6‐VSTA. This enhanced substrate coordination in the immediate vicinity to the active site might be one cause for the improved PET degradation of PET6‐VSTA. The experimentally shown significant increase in PET degradation induced by two small mutations in PET6‐VSTA further underpins the evolutionary potential of PET6.

To date, there is some crystallographic data on the substrate interaction of PET degrading enzymes, however they rely on shorter substrate analogues instead of the PET polymer.^16^, ^55^, ^56^, ^57^ In order to circumvent this limitation, MD simulations can be employed, as done here. The value and significance of this method has been demonstrated by several studies that successfully generated improved variants of PET degrading enzymes using MD simulations.^14^, ^58^, ^59^, ^60^ These studies not only confirm a mode of interaction comparable to the one we observe, with a strong proportion of aromatic interactions and simultaneous participation of polar interactions, but also illustrate for example the diverse binding of the substrate by means of several binding modes.^16^

## 3 CONCLUSION

The enzyme PET6 that we investigated in this study shows a fascinating adaptation toward its saline environment. Sodium and chloride ions stabilize the protein and decisively promote its activity. While the activity profile strongly differs from IsPETase, similar trends have been observed for other PET hydrolases and their interaction with divalent cations. The optimal working conditions of the enzyme were determined to be around 50°C and 1–1.5 M salt. The X‐ray structure confirmed the expected fold and stabilizing interactions but also highlighted some differences that led to testable mutants. MD simulations further revealed a stable interaction between enzyme and substrate with specific molecular contacts positioning the substrate appropriately toward the active site. The comparison of the double mutant additionally provided insights into how the structural changes translate to an increased activity.

An ongoing discussion about PET degrading enzymes is their actual contribution to PET waste decomposition in the environment, which has not been assessed by any study on an experimental level yet. Based on our findings, we propose that PET6 is a worthy candidate to study this topic. The genus *Vibrio* is ubiquitous in saline environments, and we demonstrated the prevalence of PET6 homologs in several *Vibrio* species. There is also a coincidence of high plastic concentrations in salt marshes, estuaries, and oceans with the confirmed occurrence of *Vibrio* in these environments and the plastisphere itself. As recently shown by Menzel et al.^61^ even comparatively low levels of enzyme activity can significantly impact the integrity and thus the fate of PET material. Hence, a PET degradation activity of PET6 and its homologues in marine and estuarine environments seems feasible.

## 4 MATERIALS AND METHODS

### 4.1 Bioinformatics analysis

Pangenome analysis and the illustration of the corresponding differential gene expression were conducted with Anvi'o 6.1 (83). GCs were built with a minbit of 0.5.

A BLASTp search with the sequence of PET6 (WP_021018894.1) as a query was performed against all *Vibrio* proteomes (taxid 662) in the non‐redundant database from NCBI to identify closely related homologs of the PETase (query coverage >99%, sequence identity >80%). Multiple sequence alignments were carried out with T‐Coffee in Expresso mode.^62^ Maximum likelihood trees were calculated with RAxML 8.2.10^63^ with 500 bootstraps and visualized on MEGA X.^64^

The genomes of the PETase‐coding *V. gazogenes* (GCF_002196515.1), *V. palustris* (GCF_900162645.1), *V. ruber* (GCF_900163965.1), *V. spartinae* (GCF_900149295.1), and *V. zhugei* (GCF_003716875.1) in GenBank format were fetched from NCBI. After file pre‐processing with the script “anvi‐script‐process‐genbank,” a pangenome analysis was conducted with Anvi'o 7.1.^65^, ^66^, ^67^ GCs were built with a minbit of 0.5. ANI was calculated using FastANI.^68^ For the more extensive pangenomic analysis in Figure S1, the genomes of *V. fischeri* (*Aliivibrio fischeri*; GCF_000011805.1), *V. casei* (GCF_003335255.1), *V. algivorus* (GCF_007623795.1), *V. mediterranei* (GCF_002214345.1), *V. maritimus* (GCF_003263775.1), *V. splendidus* (GCF_003050125.1), *V. coralliilyticus* (GCF_013266665.1), *V. diabolicus* (GCF_011801455.1), *V. parahaemolyticus* (GCF_000196095.1), *V. hepatarius* (GCF_013114105.1), *V. harveyi* (GCF_000770115.1), *V. alginolyticus* (GCF_001471275.2), *V. natriegens* (GCF_001456255.1), *V. fluvialis* (GCF_001558415.2), *V. cholera* (GCF_000006745.1), *V. proteolyticus* (GCF_000467125.1), *V. metschnikovii* (GCF_009763765.1), *V. anguilarum* (GCF_002287545.1), *V. aerogenes* (GCF_900130105.1), *V. aestivus* (GCF_003263845.1), *V. hangzhouensis* (GCF_900107935.1), *V. mangrovi* (GCF_900184095.1), *V. nitrifigilis* (GCF_015686695.1), *V. quintilis* (GCF_900143745.1), *V. tritonius* (GCF_001547935.1), *V. vulnificus* (GCF_004319645.1), and *V. ziniensis* (GCF_011064285.1) were included and processed as described above.

### 4.2 Cloning

The *pet6* gene (residues 25–297, NCBI Ref. Seq. WP_077316261.1) was cloned into a pET28a vector (Merck Millipore Novagen) via the restriction sites *Nde*I and *Sal*I, creating a construct with a N‐terminal 6x‐HisTag upon expression, while the IsPETase gene (residues 28–290) was inserted into a pET21b vector (Merck Millipore Novagen) using *Nde*I and *Xho*I to feature a C‐terminal 6x‐HisTag upon expression. Primers for the introduction of mutations were designed with the tool NEBaseChanger (New England Biolabs), the PCR was conducted according to the parameters suggested by the design tool. After PCR cleanup, 6 μl DNA (≈50 ng/μl) was combined with 1 μl each of T4 Ligase Buffer (10×), T4 Ligase (400 U/μl), *Dpn*I (20 U/μl), and T4 PNK (10 U/μl) (New England Biolabs) and incubated for 1 hr at RT. After transformation of TOP10 cells with ligation mix, positive clones were identified by DNA sequencing (Eurofins Genomics GmbH).

### 4.3 Protein production and purification

T7 Shuffle cells (New England Biolabs) were chemically transformed with the vectors containing the genes of the enzymes; afterward, the corresponding antibiotics were constantly present in the media. Main cultures were grown in TB media at 37°C to an OD of 1.5 before the temperature was lowered to 18°C, and protein expression was induced by adding IPTG to a final concentration of 300 μM. After 18 hr, the cells were harvested by centrifugation at 5,000*g* and resuspended in IMAC binding buffer (300 mM NaCl, 50 mM phosphate, 50 mM Imidazole, pH 7.4) with 10 ml/g wet weight. After sonication, cell debris was removed by centrifugation for 1 hr at 50,000*g* and vacuum filtrated through a 0.22 μM filter. The clarified lysate was loaded onto a Cytivia HisTrap 5 ml column. After washing with 20 column volumes (CV) binding buffer, the protein was eluted with a linear gradient (25 CV) of elution buffer (300 mM NaCl, 50 mM phosphate, 400 mM Imidazole, pH 7.4). The purification of the enzymes IsPETase and PET6 wildtype were polished with a size exclusion (SEC) run on a Cytivia Superdex 26/600 75 pg (SEC buffer: 150 mM NaCl, 25 mM HEPES, pH 7.4), while the PET6 variants were dialyzed in SEC buffer after the IMAC step. Eventually, the proteins were concentrated to 50–300 μM to prepare aliquots of 100 μl, which were flash‐frozen until further use.

### 4.4 Crystallization of PET6 and structure determination

Sitting drop vapor diffusion experiments were conducted with various premixed crystal screens, including the JCSG Core Suite from Qiagen. The crystal screening was done in Intelli 3‐well plates which were set up with a Crystal Phoenix (Art Robbins Instruments) setting drops in a 1:1 ratio with 0.4 μl each, where PET6 was used in a concentration of 12.3 mg/mL. The protein crystallized in a condition containing 0.1 M sodium potassium phosphate pH 6.2 and 35% 2‐methyl‐2,4‐pentanediol (MPD), yielding crystals suitable for X‐ray diffraction experiments without further optimization. The obtained crystals were frozen without the addition of a cryoprotectant and diffraction data was collected at the BESSY synchrotron. Two datasets from the same crystal were recorded at the beamline MX14.2 with 13.5 keV with an exposure time of 0.1 s per 0.1° and 2,000 images each. The datasets were processed with XDSAPP 2 and merged with Xdsconv. The crystallographic data quality indicators show good results throughout with the resolution cut‐off at 1.4 Å resulting in a R‐meas of 5.4% (30.0%), I/σ(I) of 19.45 (4.70), and CC1/2 of 99.9 (92.3), with the expression in brackets as the respective value in the highest resolution (Table S2). However, the moderate data completeness of 95.6% (91% in the highest resolution shell) is typical for the P1 space group. The phase was solved using Phenix Phaser employing a homology model of PET6 generated by MODELLER.^69^ Refinement and model building was done with Phenix Refine^70^ and Coot.^71^ During processing and solving of the structure, it could not be overlooked that this crystal with the triclinic space group P1 is strongly tending toward a higher symmetry, the cell parameters (*a* = 44.8, *b* = 72.6, *c* = 72.8, *α* = 119.8°, *β* = 91.6°; *γ* = 91.8°) are very close to a hexagonal crystal system with theoretical cell dimensions *a* = *b* ≠ *c* and *α* = *β* = 90°; *γ* = 120°. But processing the data sets merged or unmerged at higher symmetry did not successfully solve the structure, suggesting that higher symmetry was distorted. This could be related to the omission of cryoprotectant during the flash freezing step, only relying on the present MPD in the condition itself.

### 4.5 Activity assay on PET

The PETase activity of the enzymes was measured by incubating the enzymes in PET‐coated 96‐well microtiter plates. The coating is part of an assay platform described in detail in our previous paper.^47^ In short, commercially available post‐consumer PET (CleanPET, Veolia GmbH) is dissolved in trifluoroacetic acid and applied on 96‐well microtiter plates (Nunc 96‐well clear, Fisher Scientific), excess PET solution drained, and eventually, the plate is dried at 63°C to obtain the PET coating. For the actual experiments, each well was filled with 50 μl enzyme solution where 50 mM borate pH 8.5 was the basis while enzyme and sodium chloride concentration as well as incubation temperature and time were varied according to the individual experiment. After incubation, the solution was mixed with four parts acetonitrile containing 1% formic acid followed by centrifugation. The degradation products were analyzed using the UHPLC (Thermofisher Ultimate 3000 RS) system on a reversed phase C18 column (Kinetex 1.7 μm EVO C18, 100 Å, 50 × 2.1 mm Phenomenex). For fast separation at a flow rate of 1.3 ml/min the following multi‐slope gradient was employed starting at 100% solvent A (water + 0.1% TFA) increasing acetonitrile as solvent B in the following pattern: 0.04 min—15%, 0.4 min—20%, 0.75 min—50%, 0.95 min—80%, and 2.1 min—80%. One microliter samples were injected onto the column; absorption was measured at 240 nm at a rate of 50 Hz. Six to 30 replicates were used for every condition to calculate a mean total product release where TPA, MHET, and BHET are combined.

### 4.6 Differential scanning calorimetry

Differential scanning calorimetry (DSC) runs were done with the proteins under different buffer conditions to determine the stability and melting point of the enzymes. While the buffer basis was kept constant with 50 mM borate pH 8.5, sodium chloride concentrations were varied (50, 500, and 1,000 mM), with the optional addition of 10 mM KCl. The proteins were dialyzed extensively and prepared in a final concentration between 0.5 and 1.2 mg/mL. Before applying the sample, the instrument (Malvern Microcal PEAK DSC) was thermally equilibrated with corresponding buffer‐buffer runs; scanning range was set between 25 and 70°C with a speed of 120 K/hr.

### 4.7 Molecular dynamics simulations and binding mode analysis

The PET6 crystal structure was prepared with the program CHARMM,^72^ using the CHARMM36^73^, ^74^ force field. For the simulations we used chain A of the crystal structure and the corresponding water molecules. The mutations for the PET6‐VSTA structure were introduced with PyMOL.^75^ Disulfide bonds were set, and hydrogens were added with the HBUILD routine in CHARMM. Protonation probabilities were calculated using MEAD^76^, ^77^ and GMCT.^78^ Two hundred equilibration scans and 100,000 production scans were performed at 323 K with 1.05 M ionic strength and permittivity 4 for the protein and 80 for the solvent in the pH range 0–14 with steps of 0.25. The protonation states of titratable groups were set according to this calculation. For the MD simulations, we used a PET tetramer as ligand applying published parameters.^79^ The carboxyl ends of the ligand were modeled as methyl esters to simulate a neutral continuation of longer PET chains. The initial position of the ligand was modeled according to the inhibitor *p*‐nitrophenol bound in the crystal structure with the PDB‐ID 5XH2. Both protein structures were superimposed and the second repeat unit of the PET tetramer was superimposed with the phenyl ring of *p*‐nitrophenol. The prepared protein with ligand was solved in a cubic box of water molecules extending at least 25 Å from the protein ligand complex with an ion concentration of 1.05 M NaCl. All MD simulations were run with ACEMD^80^ at 323.15 K. In total, we performed 20 independent production runs about 50 ns for each protein whereas we just considered trajectories where the PET tetramer remained bound to the protein throughout the trajectory and where a partially bound ligand did not form self‐stacking interactions. For PET6‐wt 8 trajectories fulfilled these criteria and for PET6‐VSTA more than 8 trajectories fulfilled them. Thus, for both proteins, we analyzed 8 independent production runs each 50 ns long. The processing of the MD trajectories was performed with MD analysis.^81^, ^82^ Errors of the calculated shares of interactions were obtained by dividing each total trajectory for PET6‐wt and for PET6‐VSTA into five consecutive trajectories of equal size and calculating the standard deviation of the respective shares among them. Binding modes were obtained on the basis of the Tanimoto similarity coefficient.^83^ A coefficient of 1 means an identical interaction pattern and a coefficient of 0 means no identical interactions. Similar interaction patterns among all the structures in the trajectories can then be clustered. For that, the analysis of interactions and the Tanimoto interaction fingerprint analysis were performed with ProLIF.^52^ Clustering for the binding mode analysis was performed with the *k*_Means_ algorithm of scikit‐learn.^84^ The optimal number of clusters was obtained by the Silhouette score. As representative structure for each cluster the closest structure to the cluster center was taken.

## AUTHOR CONTRIBUTIONS

**Sebastian Weigert:** Conceptualization (equal); investigation (equal); validation (equal); visualization (equal); writing – original draft (equal); writing – review and editing (equal). **Pablo Perez‐Garcia:** Conceptualization (equal); investigation (equal); validation (equal); visualization (equal); writing – original draft (equal); writing – review and editing (equal). **Florian J. Gisdon:** Investigation (equal); validation (equal); writing – original draft (equal); writing – review and editing (equal). **Andreas Gagsteiger:** Investigation (equal); validation (equal). **Kristine Schweinshaut:** Investigation (equal). **G. Matthias Ullmann:** Investigation (equal); validation (equal); writing – review and editing (equal). **Jennifer Chow:** Conceptualization (equal); investigation (equal); writing – original draft (equal); writing – review and editing (equal). **Wolfgang R. Streit:** Conceptualization (equal); funding acquisition (equal); writing – review and editing (equal). **Birte Höcker:** Conceptualization (equal); funding acquisition (equal); investigation (equal); writing – original draft (equal); writing – review and editing (equal).

## CONFLICTS OF INTEREST

The authors declare no conflict of interest.

## Supporting information


**TABLE S1:** BLASTp searches against NCBI taxid662 Vibrio
**TABLE S2:** Quality indicators for crystallographic data and model building for the crystal structure of PET6
**FIGURE S1:** Molecular relations between the putative Vibrio PETases and all PETases in the PAZy database
**FIGURE S2:** Probability density distributions of distances related to the mutated residues Thr91 and Ala92 in PET6‐VSTA
**FIGURE S3:** Comparison of electron densities at the three proposed ion‐binding sites in PET6

## Data Availability

All data to support the conclusions of this manuscript are included in the main text and supporting information. Coordinates and structure factors have been deposited to the Protein Data Bank (PDB) with accession code: 7Z6B.
