# Supplementary material for: Investigation of the halophilic PET hydrolase PET6 from Vibrio gazogenes
Source: Protein Sci. 2022 Dec;31(12):e4500. doi: 10.1002/pro.4500 (PMC9679969; doi:10.1002/pro.4500)
Supplement: Supplementary file 1 — TABLE S1: BLASTp searches against NCBI taxid662 Vibrio TABLE S2: Quality indicators for crystallographic data and model building for the crystal structure of PET6 FIGURE S1: Molecular relations between the putative Vibrio PETases and all PETases in the PAZy database FIGURE S2: Probability density distributions of distances related to the mutated residues Thr91 and Ala92 in PET6‐VSTA FIGURE S3: Comparison of electron densities at the three proposed ion‐binding sites in PET6 [file PRO-31-e4500-s001.pdf]

Supporting Information

for

Investigation of the halophilic PET hydrolase PET6 from *Vibrio gazogenes*

Weigert, Sebastian[1]; Perez-Garcia, Pablo[2,3,]; Gisdon, Florian[1]; Gagsteiger, Andreas[1]; Schweinshaut, Kristine[1]; Ullmann, Matthias[4]; Chow, Jennifer[2]; Streit, Wolfgang[2]; Höcker, Birte[1,*]

The file includes:

Table S1: BLASTp searches against NCBI taxid662 *Vibrio*

Table S2: Quality indicators for crystallographic data and model building for the crystal structure of PET6

Figure S1: Molecular relations between the putative Vibrio PETases and all PETases in the PAZy database

Figure S3: Comparison of electron densities at the three proposed ion-binding sites in PET6

Figure S2: Probability density distributions of distances related to the mutated residues Thr91 and Ala92 in PET6-VSTA

Table S1: BLASTp search against NCBI taxid662 *Vibrio*

Description	Common Name	Max Score	Total Score	Query Cover	E value	Per. ident	Acc. Len	Accession
hypothetical protein [Vibrio gazogenes]	Vibrio gazogenes	614	614	100%	0	100,00%	297	WP_077316261.1
hypothetical protein BSQ33_03270 [Vibrio gazogenes]	Vibrio gazogenes	586	586	100%	0	94,95%	298	ASA57064.1
lipase [Vibrio gazogenes]	Vibrio gazogenes	585	585	100%	0	94,95%	297	WP_021018894.1
hypothetical protein [Vibrio spartinae]	Vibrio spartinae	584	584	99%	0	94,59%	297	WP_083602694.1
Lipase 1 [Vibrio ruber DSM 16370]	Vibrio ruber DSM 16370	558	558	99%	0	89,53%	317	SJN59255.1
alpha/beta hydrolase [Vibrio ruber]	Vibrio ruber	557	557	99%	0	89,53%	297	WP_139344129.1
alpha/beta hydrolase [Vibrio zhugei]	Vibrio zhugei	513	513	100%	0	80,81%	297	WP_123015358.1
hypothetical protein [Vibrio palustris]	Vibrio palustris	501	501	100%	5,00E-179	79,93%	299	WP_077315388.1
RICIN domain-containing protein [Vibrio gazogenes]	Vibrio gazogenes	73,9	73,9	78%	2,00E-12	26,05%	469	WP_021021550.1
RICIN domain-containing protein [Vibrio spartinae]	Vibrio spartinae	73,6	73,6	78%	2,00E-12	25,48%	469	WP_074375079.1
RICIN domain-containing protein [Vibrio spartinae]	Vibrio spartinae	72	72	78%	7,00E-12	24,71%	469	WP_182288351.1
RICIN domain-containing protein [Vibrio zhugei]	Vibrio zhugei	71,2	71,2	78%	9,00E-12	24,32%	394	WP_123015726.1
RICIN domain-containing protein [Vibrio rhizosphaerae]	Vibrio rhizosphaerae	70,1	70,1	78%	3,00E-11	24,71%	469	WP_051680565.1
RICIN domain-containing protein [Vibrio ruber]	Vibrio ruber	69,7	69,7	78%	4,00E-11	24,71%	469	WP_077337226.1
RICIN domain-containing protein [Vibrio gazogenes]	Vibrio gazogenes	69,7	69,7	78%	4,00E-11	24,71%	469	WP_072955206.1

Table S2: Quality indicators for crystallographic data and model building for the crystal structure of PET6. Statistics for the highest resolution shell are shown in parentheses.

Structure<br>(PDB ID)	PET6<br>7Z6B
Wavelength (Å)	0.9184
Resolution range (Å)	25.7 - 1.4 (1.45 - 1.4)
Space group	P 1
Unit cell<br>a, b, c (Å)<br>α, β, γ (°)	 44.82, 72.61, 72.76,<br>119.78, 91.64, 91.82
Total reflections	566942 (37590)
Unique reflections	150015 (14386)
Multiplicity	3.8 (2.6)
Completeness (%)	95.77 (91.87)
Mean I/sigma(I)	19.45 (4.70)
Wilson B-factor	9.53
R-merge	0.04679 (0.2477)
R-meas	0.05424 (0.3)
R-pim	0.0271 (0.1654)
$CC_{1/2}$	0.999 (0.928)
CC*	1 (0.981)
Reflections used in refinement	149922 (14378)
Reflections used for R-free	7493 (718)
R_{work}	0.0996 (0.1431)
R_{free}	0.1264 (0.1727)
CC_{work}	0.982 (0.956)
CC_{free}	0.975 (0.931)
Number of non-hydrogen atoms	8045
macromolecules	6815
ligands	648
solvent	960
Protein residues	831
RMS(bonds)	0.014
RMS(angles)	1.52
Ramachandran favored (%)	98.18
Ramachandran allowed (%)	1.82
Ramachandran outliers (%)	0.00
Rotamer outliers (%)	0.66
Clashscore	6.67
Average B-factor	15.68
macromolecules	13.05
ligands	36.27
solvent	28.56

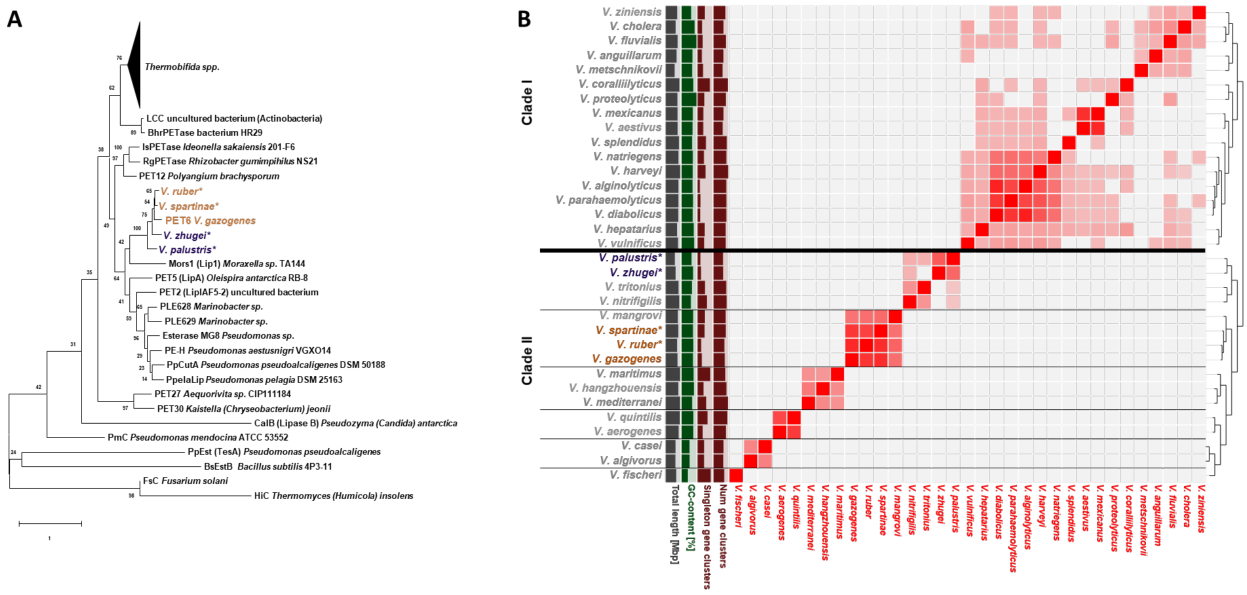

Figure S1: Molecular relations between the putative Vibrio PETases and all PETases in the PAZy database (A). Pangenome analysis of 33 Vibrio genomes (B). The genomes of 28 pathogenic and non-pathogenic Vibrios (grey) that do not code for a PET6 homolog were compared against the ones in the pangenomic analysis in Figure 1. The analysis includes 27,156 gene clusters (GCs) involving 135,122 individual gene calls. Total length (range: 0-6 Mbp), GC content (30-50 %), number of singleton GCs (0-1,000) and number of gene clusters (0-5,000). Genomes are arranged according to ANI of the aligned fraction (red), which reveals two major clades (left). *V. fischeri* was renamed to *Aliivibrio fischeri*.[1]

Reference:

(1) Urbanczyk, H.; Ast, J. C.; Higgins, M. J.; Carson, J.; Dunlap, P. V. Reclassification of Vibrio Fischeri, Vibrio Logei, Vibrio Salmonicida and Vibrio Wodanis as Aliivibrio Fischeri Gen. Nov., Comb. Nov., Aliivibrio Logei Comb. Nov., Aliivibrio Salmonicida Comb. Nov. and Aliivibrio Wodanis Comb. Nov. *International Journal of Systematic and Evolutionary Microbiology* **2007**, *57* (12), 2823–2829. https://doi.org/10.1099/ijs.0.65081-0.

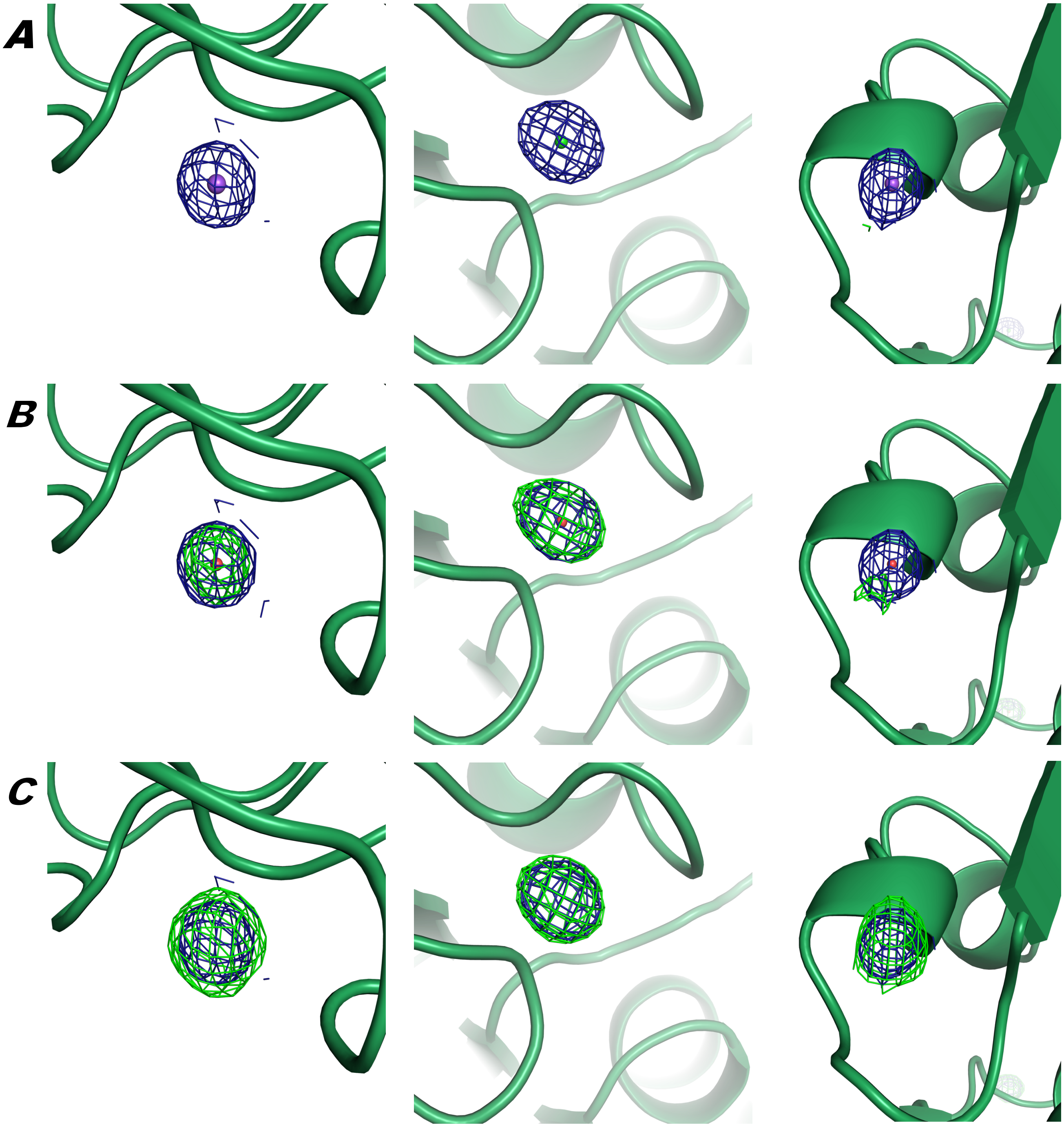

Figure S2: Comparison of electron densities at the three proposed ion-binding sites in PET6 (see same arrangement from left to right in Figure 3 D). The ions in the otherwise finalized structure were either substituted or deleted and the electron density was then recalculated: **A** for the coordinated sodium (violet) and chloride (neon green) ions, **B** when replaced by a water (red) at the coordination site, and **C** when no coordinated atoms are modelled. Densities are shown around 1.6 Å of the respective ion and are contoured at σ = 1.0 for regular density (blue) and σ = 3.0 for differential density (neon green), respectively.

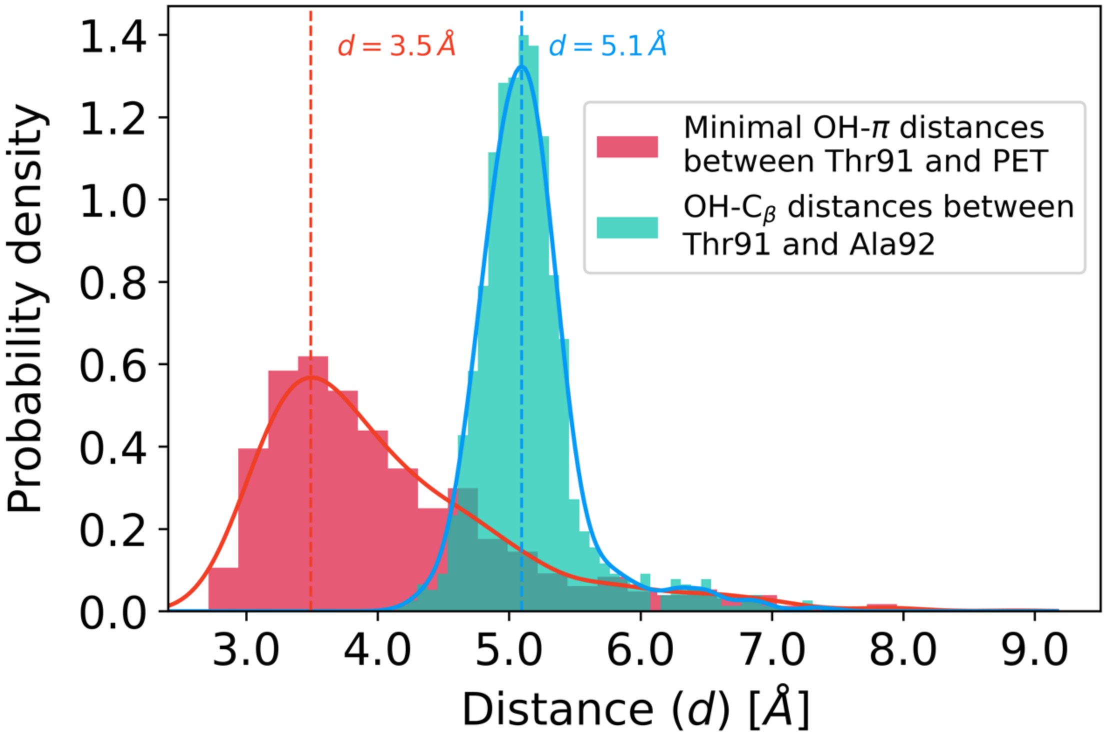

Figure S3: Probability density distributions related to the mutated residues Thr91 and Ala92 in PET6-VSTA. Red: the minimal distances between the Hγ of the hydroxyl group of Thr91 and the centroid of any π system of the PET tetramer. Blue: the distances between the Hγ of the hydroxyl group of Thr91 and the Cβ of Ala92. The respective estimated probability density functions are represented with solid lines, the respective maxima of the distributions (maximum of the respective probability density function) are represented with dashed lines. The probability density function was calculated with the Scipy Python package (scipy.stats.norm object). The optimal number of bins was calculated with the Freedman-Diaconis rule.
